# Factors associated with the delivery of specific physical activity advice to patients with non-communicable diseases by primary care physicians in Klang Valley, Malaysia: a cross-sectional study

**DOI:** 10.51866/oa.754

**Published:** 2025-02-21

**Authors:** Hakimah Khani Suhaimi, Siti Fatimah Badlishah-Sham, Ahmad Taufik Jamil

**Affiliations:** 1 MBBCh BAO, MMed Fam Med, Department of Primary Care, Medicine Faculty of Medicine, Universiti Teknologi MARA Sg Buloh Campus, Jalan Hospital, Sungai Buloh, Selangor, Malaysia. Email: sfatimah31@uitm.edu.my; 2 MBBS, MMed Fam Med, Klinik Kesihatan Seri Kembangan Jalan Besar, Taman Muhibbah, Seri Kembangan, Selangor, Malaysia.; 3 MD, Master of Community Health, Science, Department of Public Health, Medicine Faculty of Medicine, Universiti Teknologi MARA Sg Buloh Campus, Jalan Hospital, Sungai Buloh, Selangor, Malaysia.

**Keywords:** Exercise, Noncommunicable diseases, Primary health care, Physicians, Counseling

## Abstract

**Introduction:**

Specific physical activity advice delivered to patients with non-communicable diseases (NCDs) improves physical activity levels and health outcomes. This study aimed to develop a physical activity advice tool and determine the physical activity level of primary care physicians, prevalence of physical activity advice delivered to patients and its associated factors.

**Methods:**

During phase 1 of the study, a valid and reliable tool was developed to assess physical activity advice delivered by primary care physicians. Phase 2 was a cross-sectional study conducted at 12 primary care clinics using an online questionnaire assessing sociodemographic characteristics, physical activity level (Global Physical Activity Questionnaire) and physical activity advice delivered. Multiple logistic regression was used to identify the factors associated with specific physical activity advice delivered.

**Results:**

More than half of the primary care physicians (53.7%) were physically inactive. Most (79.3%) delivered specific physical activity advice to their patients. The primary care physicians who were women (odds ratio [OR]=4.54, 95% confidence interval [CI] = 1.78, 11.56), possessed postgraduate qualifications (OR=6.72, 95% CI=1.48, 30.51), received formal training in physical activity advice (OR=2.79, 95% CI=1.01, 7.79) and were physically active (OR=2.67, 95% CI=1.17, 6.10) were more likely to deliver specific physical activity advice.

**Conclusion:**

Primary care physicians should be encouraged to pursue postgraduate studies, be given training in how to deliver physical activity advice and be physically active to be able to deliver specific physical activity advice to patients seen in NCD clinics.

## Introduction

Physical inactivity is a global public health concern, with 28.3% of all adults failing to reach the recommendations for physical activity set by the World Health Organization (WHO).^1^ The overall prevalence of physical inactivity recorded among Malaysian adults was 43.7% in 2006,^2^ which improved to 29.9% in 2023.^3^ This may be attributed to the implementation of the National Strategic Plan for Active Living by various government agencies to encourage Malaysians to be more active.^4^ However, almost half (45.6%) of Malaysian primary healthcare workers are reported to be physically inactive, a proportion larger than that of the general population.^5^

Physical inactivity contributes to the rising prevalence of non-communicable diseases (NCDs), resulting in premature mortality and economic burden.^6^ Physical activity advice should be delivered as part of primary and secondary prevention of NCDs. According to the 2019 National Health Morbidity Survey, an adult person sees their primary care physician to seek treatment on an average of 3.54 visits yearly.^7^ Primary care physicians should utilise this opportunity to identify physically inactive patients and provide relevant physical activity advice.^8,9^

The delivery of physical activity advice is a non- pharmacological treatment aiming to motivate patients to reach the recommended physical activity level. Primary care physicians’ physical activity advice has a positive impact on patients’ physical activity levels and health outcomes^10,11^ such as reduction in weight and triglyceride level and increment in total energy expenditure.^11^

The frequency of physical activity advice delivered by primary care physicians during consultation ranges from 30.9% to 100%.^12-15^ There is a wide spectrum of how physical activity is delivered, ranging from primary care doctors briefly mentioning general physical activity recommendations to customised or individualised specific physical activity advice tailored to each patient.^16^ Although general advice on physical activity may require less time and effort, specific physical activity advice has been shown to improve the physical activity levels of patients.^17,18^ For the purpose of this study, specific physical activity advice was defined as providing specific information on physical activity recommendations according to the well-established guidelines including the frequency, intensity, time (or duration) and type principle.^19^ Multiple factors influence primary care physicians’ delivery of physical activity advice including sociodemographic characteristics, personal physical activity levels and knowledge, confidence and perceived barriers in physical activity counselling.^12-15^

To our knowledge, there is scarce published literature assessing the prevalence of specific physical activity advice delivered by primary care physicians to their patients with NCDs and its associated factors. A randomised controlled trial conducted in a university primary care clinic evaluated the effects of a patient- centred assessment and counselling for exercise intervention among sedentary male patients aged 30-65 years. However, the study did not directly assess the prevalence of the types of physical activity advice delivered by primary care physicians, as the exercise counselling delivered was pre-determined and standardised.^11^ Another local study assessed the frequency of initiation of exercise counselling among patients with chronic diseases but did not evaluate the types and depth of exercise counselling delivered.^15^

To date, there are no available validated tools for assessing the types of physical activity advice delivered. Therefore, this study aimed to i) develop and validate a tool for measuring the types of physical activity advice delivered by primary care physicians, ii) determine the personal physical activity levels of primary care physicians and iii) evaluate the types of physical activity advice delivered to patients and their associated factors. The outcomes of this study can guide stakeholders in designing interventions needed to improve the delivery of specific physical activity advice by primary care physicians to their patients.

## Methods

This study was conducted in two phases. In phase 1, a tool was developed and validated to assess the types of physical activity advice delivered by primary care physicians. Phase 2 was a cross-sectional study aiming to determine the personal physical activity levels, types of physical activity advice delivered by primary care physicians and their associated factors.

### Phase 1

An extensive literature review on the spectrum of the types of physical activity advice was conducted to determine its conceptualisation.^20^ Physical activity was defined as any bodily movement produced by the skeletal muscles requiring energy expenditure including activities undertaken while working, playing and carrying out household chores.^21^ Meanwhile, exercise was described as a sub-category of physical activity that is planned, structured and repetitive.^21^ The main framework used as a reference was the WHO Guidelines on Physical Activity and Sedentary Behaviour.^22^ Upon identification of the core components of the types of physical activity advice, the most appropriate question format was determined as closed-ended multiple-choice questions (MCQs). A single domain was labelled ‘physical activity advice’, and item generation was then conducted.

The items generated underwent content validation to assess the relevance of the items using the item-level content validity index (I-CVI). Content validation was conducted by an expert panel, which consisted of four family medicine specialists (FMSs), a sports medicine specialist and a public health specialist who had vast experience with physical activity in healthcare. The expert panel scored each item from 1 (‘item not relevant’) to 4 (‘item very relevant’). Any items with an I-CVI of <0.83 were omitted from the questionnaire.^23^

The MCQs then underwent face validation by 10 primary care physicians to assess their clarity and comprehensibility. They judged the clarity of each item from a score of 1 (‘item not clear’) to 4 (‘item highly clear’). The acceptable item-level face validity index ( I-FVI) was set at a minimum of 0.83.^24^ A pilot study was conducted among 30 primary care physicians in a university primary care clinic and at Klinik Kesihatan Taman Ehsan to assess the operational characteristics via an online questionnaire. An invitation link was sent to primary care doctors through their FMSs. Test-retest reliability analysis with Kappa statistics was performed to assess the stability of the physical activity advice items by redistributing the same questionnaire to participants 2 weeks following their first response.

### Phase 2

A cross-sectional study was conducted among primary care physicians practising in 12 urban government primary care clinics within Klang Valley. Primary care physicians (either FMSs or medical officers) who were fully registered with the Malaysian Medical Council, had more than 1 year of working experience in a primary care clinic and consented to participate in the study were included. Primary care physicians who were on long leave for more than 6 months and those who did not routinely provide physical activity advice when seeing patients with NCDs were excluded. The sampling method was convenience sampling.

The sample size was calculated based on the study conducted by O’Brien et al., who found that 86.2% of participants provided verbal physical activity counselling to their patients.^13^ Using the single-proportion formula based on an a-value of 0.05 and an absolute precision of 5.0%, we calculated the minimum sample size as 183. Given a 50% non-response rate to the online questionnaire,^25^ the link to the questionnaire was distributed to at least 366 primary care physicians.

The questionnaire used in this study consisted of three sections: i) sociodemographic characteristics, ii) physical activity advice tool (developed from phase 1 of the study) and iii) the Global Physical Activity Questionnaire (GPAQ). The sociodemographic data collected included age, sex, race, duration of practice in primary care, place of practice, type of clinic (family doctor concept [FDC] clinic/non-FDC clinic), average patient load per day, average consultation time per patient, highest qualification and formal training received in physical activity.

The GPAQ was developed in 2002 and was proven to be valid and reliable.^26^ It is a selfreported instrument based on a person’s typical or usual weekly pattern. The tool comprises 16 questions divided into three domains: i) activity at work, ii) travel to and from places and iii) recreational activities. The GPAQ uses metabolic equivalents (METs) to express the intensity of physical activity. Respondents are categorised as either physically active if the total MET minutes per week is ≥600 or physically inactive if the total MET minutes per week is <600.

Data were collected from November 2021 to February 2022 using the online questionnaire. The link to the online questionnaire was sent to the FMSs of the selected clinics via WhatsApp, who then distributed the link to the primary care physicians within the clinics. Primary care physicians who wished to participate in the study were required to tick the consent box. Those who fulfilled the study inclusion criteria were able to proceed to the three sections of the questionnaire. Once participants completed the questionnaire, they were required to click on the submission button. Participants who were involved in phase 1 of the study were not invited to participate in phase 2 (mutually exclusive).

Data entry and statistical analysis were performed using the IBM Statistical Package for the Social Sciences version 28 (IBM Corp., Armonk, NY, USA).^27^ Descriptive statistics were used to describe the sociodemographic characteristics, physical activity levels and physical activity advice delivered. Means with standard deviations were used to describe continuous data with a normal distribution and medians with interquartile ranges (IQRs) to present continuous data with a non-normal distribution. Conversely, frequencies and percentages were utilised to describe categorical data.

Inferential analysis using simple logistic regression (SLogR) was performed, and variables with a P-value of <0.25 in this analysis were included in the multiple logistic regression (MLogR) analysis using the backward logistic regression method to adjust for any confounding factors. Model fitness (Hosmer- Lemeshow goodness-of-fit test), interactions, multicollinearity and assumptions were checked. Statistical significance was set at P<0.05, and the factors associated with the delivery of specific physical activity advice were expressed as adjusted odds ratios.

## Results

### Phase 1

In the content validation, all three items in the single domain achieved an I-CVI of 0.83-1 and were thus deemed relevant. The final I-FVI was 0.9 for all items following face validation. The final items in the MCQs following content and face validation are shown in Table 1.

**Table 1:** Multiple-choice questions following content and face validation in phase 1 of the study.

***Types of physical activity advice in primary care settings*** I would like to ask about your routine practice in the primary care clinic in giving physical activity advice when seeing patients with NCDs (e.g. obesity, hypertension or diabetes mellitus). Physical activity advice is a non-pharmacological treatment that is provided to patients with the aim of motivating them to reach the recommended physical activity level. It is given based on recommendations from established guidelines such as the Global Action Plan on Physical Activity 2018-2030 by the WHO and Garis *Panduan Aktiviti Fizikal Malaysia by Kementerian Kesihatan Malaysia.* Adults aged 18-64 years are recommended to do at least: • 150-300 minutes of moderate-intensity physical activity per week OR • 75-150 minutes of vigorous-intensity aerobic physical activity per week OR An equivalent combination of moderate- and vigorous-intensity activities throughout the week.
QUESTION 1: When seeing patients with NCDs in your general primary care clinic, how do you routinely deliver physical activity advice? (Please choose **ONE [1]** option only)
Option (a) I routinely deliver general physical activity advice that is not a specific recommendation according to established physical activity guidelines. E.g. ‘You need to exercise more’. ‘You need to be more active’. Option (b) I routinely deliver specific physical activity advice according to recommendations from established physical activity guidelines. E.g. ‘You need to do moderate-intensity exercise for at least 30 minutes, five times a week, such as brisk walking’.
***If you chose option (a) in QUESTION 1, please proceed straight to Section C of the questionnaire (GPAQ).*** ***If you chose option (b) in QUESTION 1, please continue to QUESTION 2.***
QUESTION 2: Please tick the components of the FITT principle that you routinely deliver as part of your physical activity advice to your patients (you may tick more than one option): □ Frequency of physical activity □ Intensity of physical activity (low/moderate/high) □ Type of physical activity (e.g. walking, swimming or cycling) □ Time (duration) of physical activity

NCD: non-communicable disease, GPAQ: Global Physical Activity Questionnaire, FITT: frequency, intensity, type and time

A total of 32 primary care physicians completed the questionnaire during the pilot testing. The online questionnaire took about 10 minutes to be completed. A total of 20 participants completed the questionnaire for the test-retest reliability analysis at a 2-week interval. The Kappa value was 0.737 and 0.634 for questions 1 and 2, respectively, which showed substantial agreement.

### Phase 2

**Figure 1.**
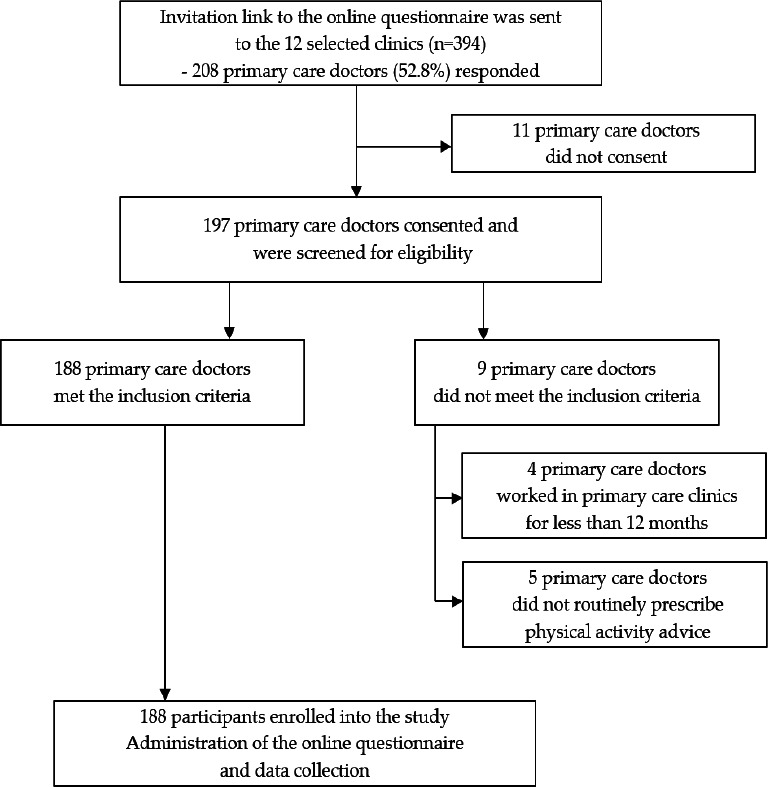
Flow chart of patient recruitment.

A total of 208 participants responded to the online questionnaire, yielding a response rate of 52.8%. However, only 188 participants fulfilled the inclusion criteria and consented to the study (Figure 1). The median age of the participants was 35 (IQR=6) years. The majority of the participants were women (80.3%) and of Malay ethnicity (63.3%). Most had neither postgraduate qualifications in family medicine (78.7%) nor formal training in physical activity advice (71.8%). The sociodemographic details are shown in Table 2.

**Table 2:** Sociodemographic characteristics.

Characteristic	Frequency (%) N=188	Median (IQR)
Age (year)		35 (6)
**Sex**		
Male	37 (19.7)	
Female	151 (80.3)	
**Ethnicity**		
Malay	119 (63.3)	
Chinese	25 (13.3)	
Indian	41 (21.8)	
Others	3 (1.6)	
**Place of practice**		
Klinik Kesihatan Cheras	26 (13.8)	
Klinik Kesihatan Seri Kembangan	24 (12.8)	
Klinik Kesihatan Jinjang	24 (12.8)	
Klinik Kesihatan Tanglin	24 (12.8)	
Klinik Kesihatan Kelana Jaya	19 (10.1)	
Klinik Kesihatan AU2	18 (9.6)	
Klinik Kesihatan Kota Damansara	17 (9.0)	
Klinik Kesihatan Dato’ Keramat	16 (8.5)	
Klinik Kesihatan Rawang	8 (4.3)	
Klinik Kesihatan Cheras Baru	6 (3.2)	
Klinik Kesihatan Setapak	4 (2.1)	
Klinik Kesihatan Seksyen 7	2 (1.1)	
**Family doctor concept clinic**		
Yes	103 (54.8)	
No	85 (45.2)	
**Postgraduate qualification**		
Without postgraduate qualification	148 (78.7)	
With postgraduate qualification	40 (21.3)	
**Received formal training in physical activity advice**		
Yes	53 (28.2)	
No	135 (71.8)	
Duration of practice (year)		6 (6)
Number of patients seen per day		30 (17)
Consultation time per patient (minute)		10 (5)

IQR, interquartile range

Slightly more than half (53.7%) (95% CI=0.47, 0.61) of the participants reported that they were physically inactive (<600 MET minutes/week). Most (79.3%) (95% CI=0.73, 0.85) delivered specific physical activity advice. Among the participants who delivered specific physical activity advice, half (52.7%) delivered all four of the FITT components.

In the SLogR analysis, the following seven variables had a P-value of <0.25: age, female sex, non-FDC clinic, postgraduate qualification, formal training in physical activity advice, duration of practice and personal physical activity level. These factors were then included in the MLogR analysis. Table 3 shows the four factors found to be significantly associated with delivering specific physical activity advice. The participants who were women, had postgraduate qualifications, received formal training in physical activity advice and were physically active were more likely to provide specific physical activity advice. The Hosmer-Lemeshow goodness-of-fit test value was not significant (P=0.34), indicating that the model fitted the data well.

**Table 3:** Simple and multiple logistic regression analyses of the association of the sociodemographic characteristics and physical activity level with the delivery of specific physical activity advice.

Variable	Simple logistic regression				Multiple logistic regression			
	Beta (SE)	Wald (df)	Crude OR (95% CI)	P-value	Adjusted beta (SE)	Wald (df)	Adjusted OR (95% CI)	P-value
**Age (year)**	0.84	4.60 (1)	2.32 (1.08, 4.99)	**0.032**	-	-	-	-
**Sex**								
Male			1				1	
Female	0.96	5.55 (1)	2.60 (1.18, 5.77)	**0.018**	1.51	10.06 (1)	4.54 (1.78, 11.56)	**0.002**
**Ethnicity**								
Malay			1		-	-	-	-
Chinese	0.49	0.15 (1)	1.63 (0.14, 18.60)	0.696	-	-	-	-
Indian	1.3	0.90 (1)	3.67 (0.25, 53.83)	0.343	-	-	-	-
Others	0.89	0.47 (1)	2.43 (0.19, 30.63)	0.493	-	-	-	-
**Family doctor concept clinic**								
Yes			1		-	-	-	-
No	0.44	1.47 (1)	1.55 (0.76, 3.15)	**0.226**	-	-	-	-
**Postgraduate qualification**								
Without postgraduate qualification			1				1	
With postgraduate qualification	1.85	6.06 (1)	6.33 (1.46, 27.54)	**0.014**	1.91	6.08 (1)	6.72 (1.38, 30.52)	**0.014**
**Received formal training in physical activity advice**								
No			1				1	
Yes	0.93	3.79 (1)	2.53 (0.99, 6.46)	**0.051**	1.03	3.92 (1)	2.79 (1.01, 7.69)	**0.048**
**Duration of practice**	0.11	3.84	1.11 (1.00, 1.23)	**0.05**	-	-	-	-
**Number of patients seen per day**	0.01	0.02 (1)	1.00 (0.97, 1.04)	0.903	-	-	-	-
Consultation time per patient (minute)	0.03	0.93 (1)	1.03 (0.97, 1.09)	0.335	-	-	-	-
**Physical activity level**								
Inactive			1				1	
Active	0.68	3.25 (1)	1.97 (0.94, 4.13)	**0.071**	0.98	5.41 (1)	2.67 (1.17, 6.10)	**0.020**
**Sedentary level**								
Sedentary			1		-	-	-	-
Not sedentary	-0.23	0.42 (1)	0.79 (0.39, 1.61)	0.519	-	-	-	-
The model fitted well (χ^2^=25.98, df=4, N=188, P<0.005). Specificity of 10.3%, sensitivity of 98% ROC=0.76, 95% CI=0.67, 0.84, P<0.001 The model assumptions were met; there was no significant interaction, multicollinearity or outlier.								

SE: standard error, OR: odds ratio, CI: confidence interval

## Discussion

To the best of our knowledge, this is the first study that developed a validated and reliable physical activity advice tool to assess the types of physical activity advice routinely delivered by primary care physicians to their patients in NCD clinics. During the content validation with the expert panel, the FITT components were added as examples to further clarify the ‘specific physical activity’ item. This is supported by the recommendations from the American College of Sports Medicine to incorporate the FITT principle into specific physical activity advice delivered to patients.^19^

This study found that slightly more than half of the primary care physicians were physically inactive. This proportion is larger than that in the study by Abu Saad et al. in which 45.6% of Malaysian primary healthcare workers were reported as physically inactive.^5^ The increased prevalence of physical inactivity among primary care physicians could be attributed to the restricted movement for outdoor recreational activities imposed by the movement control order (MCO) during data collection. The MCO was implemented nationwide as a strategy to curb the COVID-19 pandemic from 19 March 2020 to 31 December 2021. Movement restrictions were associated with lower physical activity levels lasting beyond the period of strict restrictions.^28^

In the present study, most primary care physicians delivered specific physical activity advice to their patients with NCDs to suit patients’ lifestyles and health requirements as recommended by guidelines.^19^ This is a reflection of the initiative by primary care physicians and the familiarity they have with the FITT criteria when delivering physical activity advice.

The female primary care physicians in this study were found to be more likely to provide specific physical activity advice than their male counterparts, which is similar to previous reports showing that female primary care physicians were more likely to provide physical activity counselling.^10,13^ This is due to the difference in the prioritisation of prevention between the two sexes, wherein female doctors are shown to devote more time to preventive services and psychosocial counselling.^29^

The primary care physicians who possessed postgraduate qualifications were more likely to provide specific physical activity advice to patients with NCDs than those who did not possess such qualifications. Similarly, Selvaraj and Abdullah found that primary care physicians with postgraduate qualifications were more likely to initiate physical activity counselling for patients with hypertension.^15^ The higher levels of knowledge and confidence among doctors with postgraduate qualifications may contribute to the significant difference in the delivery of specific physical activity advice to their patients.

The primary care physicians who received formal training in physical activity advice were more likely to provide specific physical activity advice than their counterparts. This is supported by a study in Brazil that reported a lack of adequate training as one of the barriers to providing physical activity counselling or advice.^12^ In contrast, O’Brien et al. found that primary care physicians provided physical activity counselling despite an overall lack of training hours in physical activity advice at both undergraduate and postgraduate levels.^13^ Formal training sessions for primary care physicians on how to deliver physical activity advice to patients with NCDs should be incorporated into continuous medical education programmes in clinics. At present, as the evidence on the effectiveness of such a strategy may be conflicting,^12,13^ a randomised controlled trial is required to prove its value.

Lastly, the primary care physicians who were physically active were more likely to provide specific physical activity advice than those who were physically inactive. This finding is comparable to previous reports whereby physically active doctors were more likely to provide physical activity counselling.^13,15^ Physically active primary care physicians are more convinced about the importance and benefits of being physically active and thus emphasise such to their patients by providing specific physical activity advice. Further, these doctors may already be in the action and maintenance stages of change themselves and are therefore motivated to influence the physical activity level of their patients.

### Strengths, limitations and implications for future research

The main strength of this study is that it developed a valid and reliable tool to determine the prevalence of physical activity advice routinely delivered to patients with NCDs. Nonetheless, this study has a few limitations. The study design employed was cross-sectional, which could determine the relationship between the variables but not the causal effects. Response bias may also occur, whereby primary care physicians who were physically active may be more interested in participating in this study, thus influencing the overall prevalence of specific physical activity counselling. Social desirability bias may also occur in that participants may respond to the questionnaire based on what they perceive as favourable to others. Another limitation of the study is the employment of convenience sampling, which may yield sampling bias. Further research should employ randomised sampling and include a larger, more sex-balanced sample to ensure the generalisability of the findings to the Malaysian setting. Direct observation during patient consultation may provide a more accurate result.

## Conclusion

Strategies should be undertaken to encourage primary care physicians to pursue postgraduate studies and develop formal training sessions to enhance the knowledge, attitudes and counselling skills of primary care physicians to deliver physical activity advice. Physical activity programmes should also be conducted among primary care physicians to increase their physical activity levels. This will be in line with the aspirations of the Ministry of Health’s National Strategic Action Plan for Active Living 20162025 of promoting physical activity to patients in a holistic approach to reduce the disease burden in Malaysia.30

## Data sharing statement

Data are kept at the Department of Primary Care Medicine, University Teknologi MARA in Selangor, Malaysia. The data presented in this study may be shared upon reasonable request and are not publicly available due to data protection laws and regulations.

**How does this paper make a difference in general practice?**

- This study developed a validated and reliable multiple-choice question-based tool to determine the types of physical activity advice delivered to patients in non-communicable disease clinics by primary care physicians.
- Physically active primary care physicians are found to deliver specific physical activity advice to their patients.
- Primary care physicians should be encouraged to be physically active, pursue postgraduate studies and receive formal training in the delivery of physical activity advice.
